# Does mental imagery make a difference in virtual reality simulation training? Results of a randomized controlled trial

**DOI:** 10.1007/s00464-025-12012-9

**Published:** 2025-08-20

**Authors:** Moritz B. Sparn, Dimitrios Chatziisaak, Rene Warschkow, Bruno Schmied, Dieter Hahnloser, Stephan Bischofberger

**Affiliations:** 1 Department of Surgery, Kantonsspital St. Gallen, 9007 St. Gallen, Switzerland; 2 Department of Visceral Surgery, University Hospital Lausanne, Rue du Bugnon 46, 1011 Lausanne, Switzerland

**Keywords:** Virtual reality simulation training, Mental training, Simulation, Laparoscopic surgery training, Surgical training, Surgical education

## Abstract

**Purpose:**

To assess the effect of mental imagery (MI) on the performance in Virtual Reality Simulation Training (VRST) and laparoscopic cholecystectomy training with a box-trainer.

**Methods:**

We randomly assigned novice laparoscopic surgeons taking part in 2023’s Davos Course to either receive MI followed by VRST vs. VRST alone. Laparoscopic performance was measured using time taken and instruments’ path lengths. We also measured surgical performance in a porcine cholecystectomy model in a box-trainer using a modified objective structured assessment of technical skills (OSATS) score. One-hundred-ten participants in the beginners’ module of the Davos Course were eligible, and all could be assessed in this study.

**Results:**

VRST performance did not differ significantly between the two groups. In the cholecystectomy exercise, participants in the MI group performed significantly better in the two subscales ‘Respect for Tissue’ and ‘Use of the Assistant’ (*p* = 0.037 and 0.035, respectively). There was no difference in the other subscales and the overall OSATS Score.

**Conclusions:**

MI did not improve young surgeons’ overall OSATS scores in a porcine laparoscopic cholecystectomy model. On two subscales of the OSATS scores that reflect skills that are hard to train with VRST, the MI group performed significantly better. Further research involving VRST is warranted to elucidate optimal training modalities.

Minimally invasive surgery (MIS) has become standard of care in many surgical procedures nowadays and laparoscopic techniques have revolutionized surgery in numerous fields [1]. The rise of robotic-assisted techniques is currently accelerating the growth of minimally invasive surgery. Acquiring the skills needed to safely perform laparoscopic procedures can be challenging. Working time restrictions, patient safety requirements and a rise in administrative tasks make it difficult to learn and teach MIS “on the job” [2, 3]. Halsted’s apprentice-tutor model, which has been in place for nearly a century, comes under growing scrutiny.

The efficacy of virtual reality simulation training (VRST) has been shown in multiple studies among other things reducing error rates and improving working speed [4–7]. VRST is important for early technical skills acquisition in laparoscopic surgery [8]. Several randomized controlled trials highlighted their growing importance as a safe, ethical, and comparable way to train basic surgical skills [7, 9, 10]. Currently, several virtual reality simulators (VRS) are available from various companies. These offer several training options and curricula including basic tasks (e.g., camera guidance, bimanual working, eye-hand coordination), advanced skills (e.g., suturing, knot-tying) and complete surgical procedures (e.g., laparoscopic cholecystectomy, appendectomy).

Mental imagery describes the process of going through a sequence of motions and recapitulating parts of or whole procedures without physically exercising them. The concept of ‘mental readiness’ has been introduced to the field of surgery by McDonald et al. that showed, that over 70% of surgeons carry out any kind of mental rehearsal before operations, and that mental readiness is considered as important as technical proficiency and much more important than physical abilities [11, 12]. Neural activation is similar between motor execution of a given task compared to MI [13]. Several studies have examined the effects of mental training, mostly in the form of mental rehearsal, on surgical performance in various tasks, with various outcomes.

The aim of this study was to evaluate the effect of a short mental imagery (MI) sequence on the performance of novice laparoscopic surgeons on different state-of-the-art commercially available Virtual Reality Simulation Trainers. Furthermore, we sought to evaluate the effect of previous MI on surgical performance in a porcine cholecystectomy model in a conventional box-trainer.

## Methods

This study was conducted as part of the 40th annual Davos Course in 2023, an international surgical training course in Davos, Switzerland (www.davoscourse.ch). The six-day course offers a blended learning experience with theoretical parts and a strong emphasis on hands-on training (open, laparoscopic & robotic).

Three different virtual reality simulators were used: LapSim®, LAP Mentor III® (Surgical Science Sweden AB, Gothenburg, Sweden), and LaparoS® (VirtaMed AG, Schlieren, Switzerland). The companies provided the simulators without any financial benefit and without any sponsoring. Employees of the companies were present during the training and experienced surgeons acted as instructors. The study protocol was written independently of the simulator companies but was approved by them before the start of the study. The simulator companies had no influence on data collection, statistical analysis nor drafting of the manuscript.

All study participants gave written informed consent to participate in the study. The Swiss Ethics Committee (Swissethics.ch) grants a general waiver for the use of purely anonymized data and an application with approval is not requested (Swiss Federal Act on “Research involving Human Beings 810.30”). All methods were carried out in accordance with relevant guidelines and regulations.

**Fig. 1:**
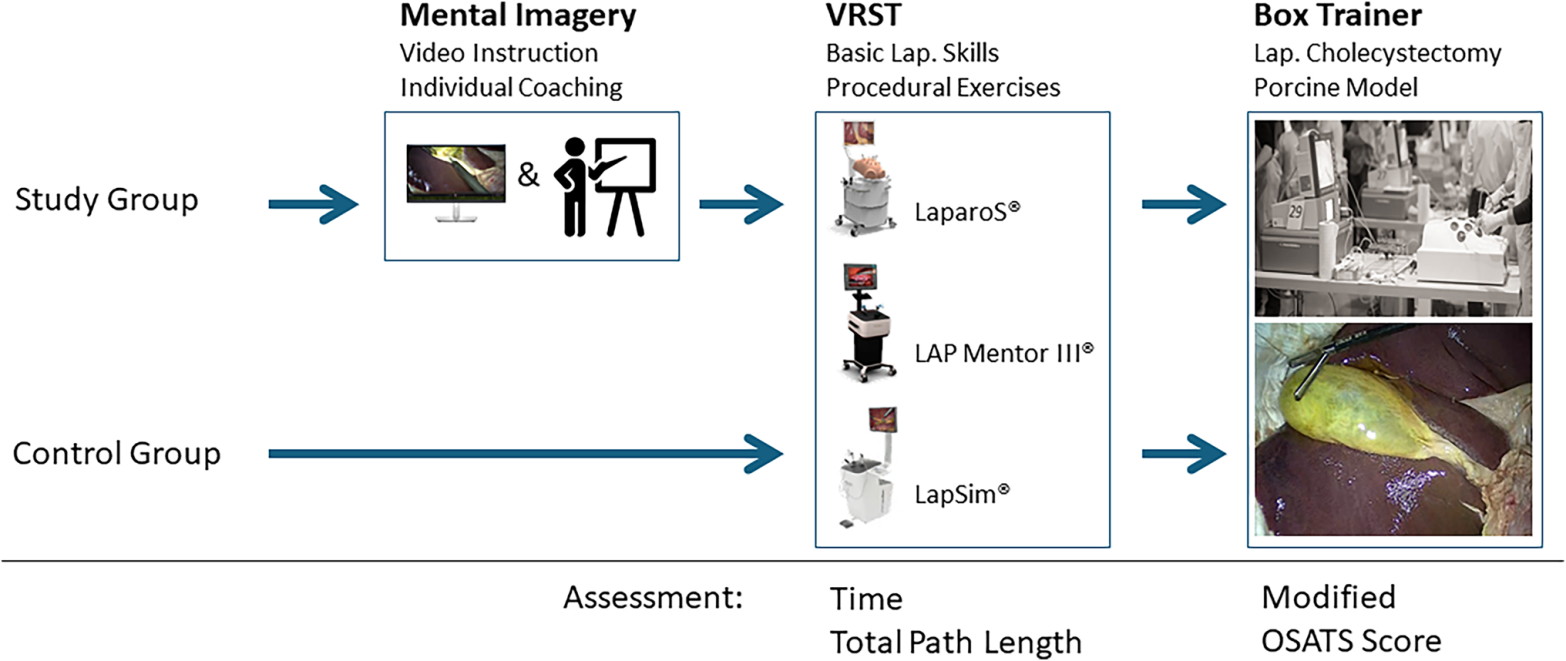
Study design

Study participants were eligible to participate in this study, if they were enrolled in the basic module of the Davos Course and gave their written consent to participate. Eligibility criteria for enrollment in the basic module were the following: clinical experience of no more than three years as a resident/surgical trainee, less than 20 appendectomies performed as the principal surgeon, less than 10 cholecystectomies performed as the principal surgeon. Participants were then randomly assigned to two groups in a 2:1 ratio in blocks of three to either undergo a mental imagery session followed by 90 min of VRST (MI group) or proceed to VRST without mental training (control group) (Fig. 1).

Participants in the MI group received a structured 15-min guided mental imagery session prior to the VR simulation training. This session was based on the PETTLEP framework (Physical, Environment, Task, Timing, Learning, Emotion, and Perspective), incorporating multisensory cues. Trainees were instructed to vividly imagine themselves performing a laparoscopic cholecystectomy, focusing on camera navigation, tissue handling, and dissection steps. An experienced laparoscopic surgeon guided the session, and participants practiced both visual and kinesthetic imagery. Technical steps of the cholecystectomy were presented according to current best practice, as taught in the SAGES Safe Cholecystectomy Program and recommended in current guidelines [14, 15]. VRST trainings consisted of basic exercises such as clipping and cutting as well as virtual reality simulations addressing specific procedures or parts of procedures (e.g., dissecting the hepatocystic triangle without ligation and cutting of the cystic structures). VRST Instructors were blinded to each participant’s study allocation.

Finally, all participants performed a laparoscopic cholecystectomy on a conventional box-trainer containing a porcine liver specimen. This exercise is the final task of the basic module of the Davos course. The primary endpoint of this study was to assess the Cholecystectomy performance. This was assessed using a modified Objective Structured Assessment of Technical Skills (OSATS) score. Assessment was carried out by experienced surgeons blinded to the participants’ allocation with a questionnaire on the following five items of the original seven items of the OSATS score: Respect for tissue, time and motion, instrument handling, knowledge of instruments and use of assistants [16]. Each item assessed using a five-point likert scale. The highest total score achievable was therefore 25 points (Appendix 1).

Secondary endpoints were procedure time and instruments’ path length on the virtual reality simulator.

For each participant, we collected the following data: demographics and previous surgical experience using a questionnaire. Furthermore, total path length of all instruments used, and time taken to complete the exercises were recorded.

Statistical analyses:

Statistical analyses were performed using a recent version of the R statistical software (www.r-project.org). A two-sided *p* value of less than 0.05 was considered statistically significant. Continuous data were expressed as the mean ± standard deviation (SD) and compared by Mann–Whitney-*U*-Test. Proportions were compared by Chi-squared statistics. The influence of MT on participants’ performance in cholecystectomy and on Time and Path Length in different VRST scenarios was assessed using mixed effects regression analyses using the R library “lme4” with MT as the fixed effect and the categorized numbers of cholecystectomies and appendicectomies performed as random effects. Internal consistency of the OSATS scores was assessed using Cronbach’s Alpha.

## Results

One hundred and ten participants took part in the study, 41 in the control group and 69 in the MI group. Participants in the control group had performed more cholecystectomies (8.4 (± 7.3) vs. 6.0 (± 3.0), *p* = 0.04) and appendicectomies (11.6 (± 13.2) vs. 7.8 (± 6.8), *p *= 0.038)) than those in the MI group.

When comparing the participants in the MI group to controls, there was no difference on the VRST exercises, except for “Hepatocystic Triangle: Variation IV” on the LaparoS®, where participants in the MI group took significantly less time to complete the task (262.6 (± 3.1) vs. 436 (± 187.8) seconds, *p* ≤ 0.001, Table 1). Otherwise, there was no statistically significant difference between MI group and control group (Table 1).

**Table 1:** Time and path length of all exercises by groups. Mixed effects model, *p* < 0.05 is considered statistically significant

Exercise name	Parameter	*N* MI group	Mean (SD) MI group	*N* controls	Mean (SD) controls	*p*-value
Cystic pedicle dissection	Time Path Length	32	287.9(138.6) 620.3(271.8)	17	263.5(55.3) 581.8(123.6)	0.479 0.573
Gallbladder resection	Time Path Length	25	569(197.7) 1110.9(360.4)	16	582.2(211.7) 1150.9(450.2)	0.836 0.747
Vascular injury	Time Path Length	28	311.9(92.1) 577.5(143)	13	297.9(76.8) 625.8(230.1)	0.624 0.397
Cystic pedicle dissection: variation IV	Time Path Length	22	401.5(132.4) 967.5(218.9)	9	417(191.2) 1066(601.1)	0.789 0.482
Hepatocystic triangle	Time Path Length	16	806.2(375.5) 1633.6(850.8)	10	950.3(347.2) 1882(732.7)	0.308 0.428
Cystic pedicle dissection: variation III	Time Path Length	13	203.3(47) 533.1(145.6)	10	222.4(74.9) 613.3(372.4)	0.432 0.456
Lap basic skills clipping and grasping	Time Path Length	14	123(19.3) 494.1(96.3)	7	134.8(31.9) 608.6(159.8)	0.265 0.030
Lap basic skills electrocautery	Time Path Length	14	287.2(67.7) 648.9(216.4)	7	270.9(93.7) 632.6(275.4)	0.629 0.876
LCHE warm-up task: clipping and cutting with two hands	Time Path Length	14	146.5(41.4) 147.8(41.7)	7	149.4(94.9) 139(64.8)	0.919 0.689
LCHE—case 1	Time Path Length	14	1036.9(371) 1544.3(429.2)	7	937.3(395.7) 1419.6(551.7)	0.551 0.548
Lap basic skills eye-hand coordination	Time Path Length	13	52.9(10.7) 248.6(61.2)	7	46.5(6) 228.8(30.1)	0.129 0.400
LCHE—case 2	Time Path Length	13	752.3(260.1) 1228.7(427.4)	6	713.4(208.9) 1218.8(399.5)	0.735 0.960
Cholecystectomy complete	Time Path Length	12	1135.4(613.8) 2403.1(1177.5)	5	1505.8(521.4) 2554.6(800.7)	0.210 0.781
Clip applying	Time Path Length	12	141.6(82.8) 301.6(283.5)	5	172.8(79.8) 321(181.9)	0.447 0.881
Coordination	Time Path Length	12	69.9(60.7) 231.1(237.3)	5	65.9(15.3) 189.1(56.1)	0.882 0.683
Cutting	Time Path Length	12	105.5(24.5) 241(124.7)	5	104.2(28.3) 202.6(60.5)	0.464 0.534
Grasping	Time Path Length	12	63.4(47.6) 382.9(283.7)	5	42.7(10.9) 281.2(52.6)	0.315 0.405
Instrument navigation	Time Path Length	12	28.2(11.2) 164(57.1)	5	28.4(12.4) 138.3(24.1)	0.999 0.318
Lifting and grasping	Time Path Length	12	108(25.1) 426.1(103.3)	5	104.2(25) 399.7(48.4)	0.817 0.566
Hepatocystic triangle: variation III	Time Path Length	8	559.9(206.2) 1163.1(472.6)	7	429.7(59.5) 752.5(197.9)	0.084 0.022
LCHE—case 3	Time Path Length	7	658.5(346.9) 1121.6(654.3)	5	661.8(328.6) 1157.2(560.5)	0.986 0.914
Hepatocystic triangle: variation IV	Time Path Length	2	262.6(3.1) 623.6(142.2)	3	436(187.8) 823.4(327.8)	< 0.001 0.005

When assessing participants’ performance in the porcine cholecystectomy, internal consistency of the OSATS scores was high. In all items and in the overall score, Cronbach’s Alpha was > 0.8 (Table 2).

**Table 2:** Internal consistency of the OSATS Score overall and for all items of the score, measured with Cronbach’s Alpha

Item	Cronbach’s Alpha (95% CI)
Overall score OSATS	0.89 (0.87–0.90)
Respect for tissue	0.88 (0.86–0.90)
Time and motion	0.84 (0.82–0.87)
Instrument handling	0.85 (0.83–0.87)
Instrument knowledge	0.88 (0.86–0.90)
Use of assistant	0.87 (0.85–0.89

*OSATS* objective structured assessment of technical skills

After correction for previous experience between MI group and control group, participants in the MI group performed statistically significantly better in the two subscales “Tissue Respect” and “Use of the Assistant”. There was no difference in the other subscales and the overall OSATS Score (Table 3).

**Table 3:** Comparison of the modified OSATS score for MT group and control group with a mixed effects regression model

Item	Effect	Estimate (95% CI)	*p*-value
Overall score	Control Group	17.40 (16.07–18.73)	
	MI Group	1.21 (0.45–2.88)	0.153
Respect for tissue	Control Group	3.53 (3.15–3.91)	
	MI Group	0.44 (0.03–0.85)	0.037
Time and motion	Control Group	3.31 (2.97–3.66)	
	MI Group	0.07 (0.36–0.51)	0.742
Instrument handling	Control Group	3.34 (3.04–3.65)	
	MI Group	0.21 (0.18–0.59)	0.294
Instrument knowledge	Control Group	3.83 (3.53–4.12)	
	MI Group	0.17 (0.20–0.54)	0.363
Use of the assistant	Control Group	3.46 (3.15–3.76)	
	MI Group	0.41 (0.03–0.80)	0.035

*OSATS* objective structured assessment of technical skills, *MI* Mental imagery

## Discussion

In this randomized controlled trial, we assessed the effect of a short mental imagery session on the performance of novice surgeons on Virtual Reality Simulators as well as on a conventional box-trainer performing a cholecystectomy. While there was no statistically significant difference in VRST, there was a tendency towards improved skills on the conventional box-trainer with significantly better OSATS scores regarding the subscales “tissue handling” and “use of the assistant”.

Mental imagery as part of mental training has proven effective in skill-learning and improving performance in music, sports and aviation [17–20].

Multiple studies examined the effect of MI on laparoscopic skills in various surgical specialties, mostly in the form of mental rehearsal, as was performed in this study. Immenroth et al. showed a significant improvement after mental training for a cholecystectomy simulation using a pelvi-trainer [21]. In another study, there was no significant impact of mental imaginery on surgical performance in a vaginal hysterectomy simulation [22]. However, a more recent systematic review and meta-analysis concludes that the overall literature on the topic “is of low quality” and that methodological differences hamper comparability of results [23]. Another recent scoping review found that mental imagery is scientifically poorly explored to date [12].

Virtual reality simulators have proven effective multiple times in learning basic laparoscopy skills as well as adopting complex surgical tasks. More than twenty years ago, the first randomized controlled trial confirmed the impact of VRST on surgical performance in cholecystectomies, in novice as well as experienced surgeons [7]. VRST has also proven effective in basic laparoscopic skills such as peg transfer and cutting exercises [24].

In this study, we measured surgical performance on a well-established box-trainer using a porcine cholecystectomy model. The comparability of this highly standardized model is a strength of this study, although the cholecystectomy examined may not directly be generalized into human procedures. There was a significant improvement in two subscales of the modified OSATS score used—’respect for tissue’ and ‘use of assistant’. In the other three subscales and overall, the MI group and control group showed equivalent performance. There are different possible explanations for this outcome. First, the items that did not show an advantage for the MI group refer to skills that are mainly taught by practice rather than understanding a concept. Second, our mental imagery session focused on procedural and safety aspects, somewhat omitting a detailed rehearsal of the complex psychomotor skills carried out during a cholecystectomy. Several other studies with rather short MT interventions also show conflicting results: In one study on knot-tying with participants with no prior experience, there was no difference with MT [25]. However, in a study on cricothyrotomy carried out by medical students with no previous surgical experience, a MT intervention as short as five minutes yielded significantly better performance of the participants compared to controls [26].

When assessing the VRST performance, MI did not significantly improve the performance of novice surgeons. This can be due to different factors: MI in our study was rather short (15 min), while in some publications, MI takes up to 150 min distributed over five consecutive days [27]. Moreover, our MI did not specifically focus on VRST exercises, but rather on a technically and manually good cholecystectomy in ‘real life’. Those procedural and safety aspects are not necessarily well trainable with VRST. Furthermore, we found a numerically small but statistically significant difference in the previous experience of the study participants. This could well have led to a distortion of the results of our study. However, since the control group had more experience than the participants in the MI group, it could also be argued that we are underestimating rather than overestimating the effect of MI. Additionally, the performance analysis was conducted using a random effects model, effectively adjusting for participants’ experience. The clinical relevance of the difference observed remains unclear, since both groups are per se on a very low level of less than 15 appendicectomies and cholecystectomies performed, respectively. In a recent study, Lombardi et al. found that residents need up to 25 cholecystectomies before they reached a stable proficiency level [28]. Therefore, none of the participants in the present study had completed their learning curve for laparoscopic cholecystectomy. Lastly, one could argue that MI as conducted in this study is rather an observational cognitive rehearsal than mental imagery. We also did not externally validate the quality of the MI video provided to study participants. Therefore, we cannot rule out the possibility that important information was missing to improve performance on the VRST. However, during assembly of the information given, we adhered to current guidelines and recommendations as described above. Furthermore, we did not perform a pre- and post-test self-assessment (e.g., Movement Imagery Questionnaire 3, revised) of the participants, therefore possibly missing a relevant difference in mental imagination at baseline [29]. This will be addressed in subsequent studies.

To date, this is the largest study that evaluates the impact of MI on VRST and surgical performance on a well-established box-trainer for cholecystectomy. Given the fact that cholecystectomies are widely considered one of the first surgical procedures young surgeons are learning, we could demonstrate that MI in combination with VRST has the potential to steepen the learning curve of novice surgeons with regard to safety-relevant aspects.

## Conclusion

MI did not improve young surgeons’ overall OSATS scores in a porcine laparoscopic cholecystectomy model. On the two subscales ‘Use of the Assistant’ and ‘Respect for Tissue’, the MI group performed significantly better. Although there was no significant effect on participants’ overall surgical performance it strengthened specifically the aspects of a cholecystectomy that are inherently hard to train on Virtual Reality Simulators. However, further research on the specific delivery of MI is needed.

## Data Availability

The datasets used and analyzed during the current study are available from the corresponding author on reasonable request.

## Appendix 1: Modified OSATS Scoring form. OSATS = objective structured assessment of technical skills

**Table:**

Evaluation of proficiency in cholecystectomy					
Name of participant			Control Nr.:		
Respect for tissue	1	2	3	4	5
	Unnecessary Force on Tissue or caused Damage by inappropriate use of Instruments		Careful Handling of Tissue but occasionally caused inadvertent Damage		Consistently handling Tissue appropriately with minimal damage
Time and motion	1	2	3	4	5
	Many unnecessary moves		Efficient Time/Motion but some unnecessary moves		Clear economy of Movement and maximum efficiency
Instrument handling	1	2	3	4	5
	Repeatedly makes tentative or awkward moves		Competent use of instruments but occasionally appearing stiff		Fluid moves, masterly use
Knowledge of instruments	1	2	3	4	5
	Frequently asking for/taking the wrong instrument		Knows names of most instruments and uses the appropriate ones		Oviously familiar with the instruments and perfect use of them
Use of assistants	1	2	3	4	5
	Consistently placing assistants poorly or failing to make use of them		Appropriate use of assistants most of the time		Strategically uses assistants to the best advantage all the time
Flow of procedure	1	2	3	4	5
	Frequently stopped and seemingly unsure of next moves		Demonstrated some forward planning with reasonable progression of procedure		Obviously planned course of procedure with effortless flow from one move to the next

## Abbreviations

MIS: Minimally invasive surgery
MT: Mental training
MI: Mental imagery
OSATS: Objective structured assessment of technical skills
VRST: Virtual reality simulation training
